# Deep Learning Based Drug Metabolites Prediction

**DOI:** 10.3389/fphar.2019.01586

**Published:** 2020-01-30

**Authors:** Disha Wang, Wenjun Liu, Zihao Shen, Lei Jiang, Jie Wang, Shiliang Li, Honglin Li

**Affiliations:** ^1^ Shanghai Key Laboratory of New Drug Design, State Key Laboratory of Bioreactor Engineering, School of Pharmacy, East China University of Science and Technology, Shanghai, China; ^2^ Research and Development Department, Jiangzhong Pharmaceutical Co., Ltd., Nanchang, China

**Keywords:** deep learning, drug metabolism, metabolites prediction, reaction rules, SMARTS

## Abstract

Drug metabolism research plays a key role in the discovery and development of drugs. Based on the discovery of drug metabolites, new chemical entities can be identified and potential safety hazards caused by reactive or toxic metabolites can be minimized. Nowadays, computational methods are usually complementary tools for experiments. However, current metabolites prediction methods tend to have high false positive rates with low accuracy and are usually only used for specific enzyme systems. In order to overcome this difficulty, a method was developed in this paper by first establishing a database with broad coverage of SMARTS-coded metabolic reaction rule, and then extracting the molecular fingerprints of compounds to construct a classification model based on deep learning algorithms. The metabolic reaction rule database we built can supplement chemically reasonable negative reaction examples. Based on deep learning algorithms, the model could determine which reaction types are more likely to occur than the others. In the test set, our method can achieve the accuracy of 70% (Top-10), which is significantly higher than that of random guess and the rule-based method SyGMa. The results demonstrated that our method has a certain predictive ability and application value.

## Introduction

**Figure 1:**
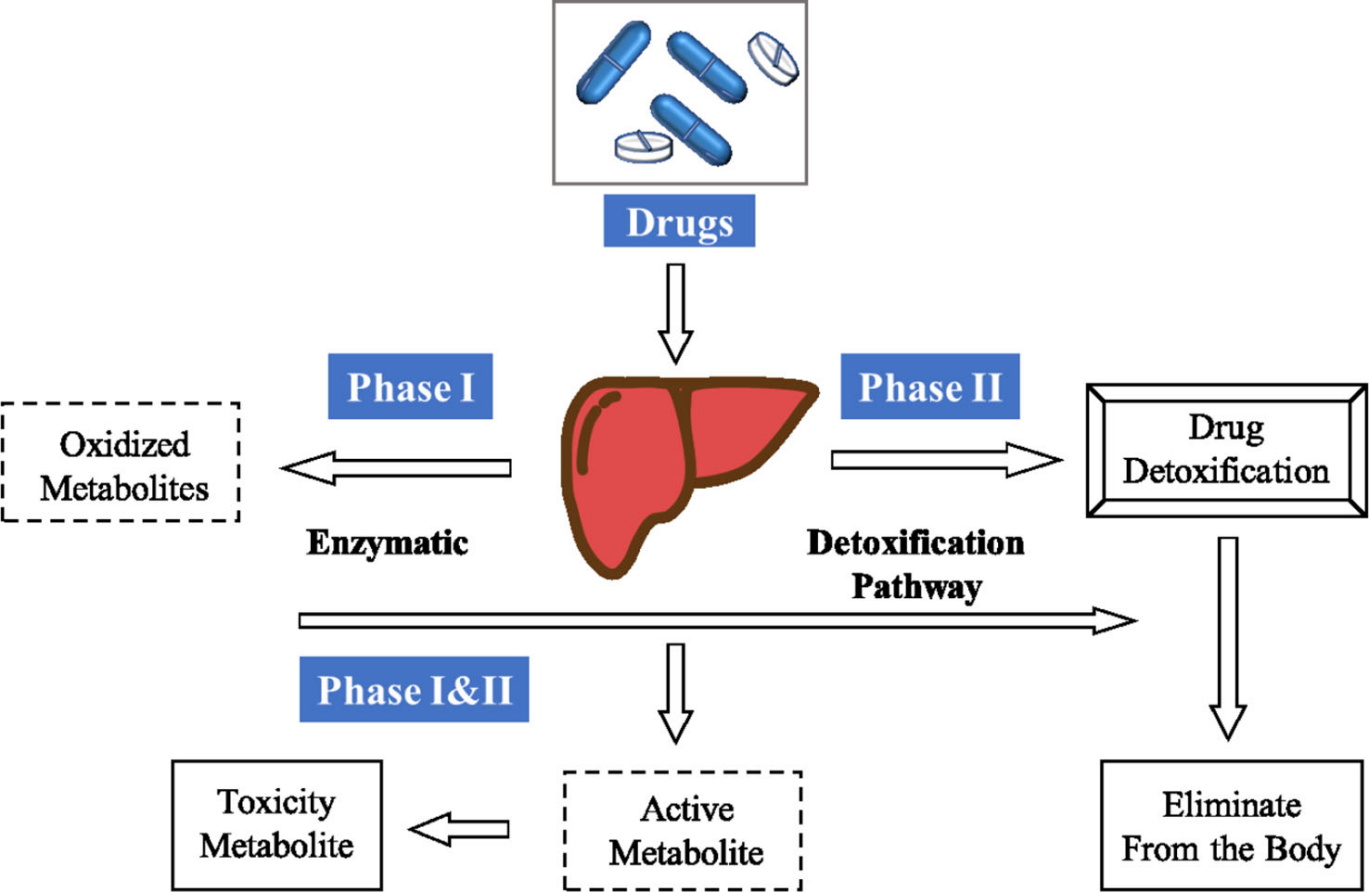
General pathway of drug metabolism.

The discovery of small molecule drugs is time-consuming, expensive and labor-intensive. (Dickson and Gagnon, 2004; Paul et al., 2010; Dimasi et al., 2015) It is resource intensive, and involves typical timelines of 10–20 years and costs that range from US$0.5 billion to US$2.6 billion (Paul et al., 2010; Avorn, 2015). In addition to economic and technical reasons, the main reason is that almost half of the candidate drugs failed in clinical trials. Up to 25% of compounds were withdrawn due to metabolic, pharmacokinetic, or toxic problems (Hwang et al., 2016). Drug metabolism can produce metabolites with physicochemical and pharmacological properties, which are significantly different from the physical and pharmacological properties of parent drugs (Kirchmair et al., 2013). As **Figure 1** shows, when drugs or other exogenous substances enter the human body, they are largely controlled by three stages of drug metabolism. In the first stage, reactive groups are introduced by oxidation, reduction, or hydrolysis. In the second stage, conjugation reactions with macromolecules occur *in vivo*. In the third stage, allogeneic and metabolites are removed from liver and intestinal cells. After these three stages, exogenous substances such as drugs may be transformed into non-physiological active substances or toxic metabolites. 70% clinical drugs are removed by the body’s metabolic system, so as part of drug development, it is also necessary to conduct in-depth research on drug metabolism (Grant et al., 2001; Embrechts and Ekins, 2007; Lazar and Birnbaum, 2012; Damsky and Bosenberg, 2012; Di, 2014; Mackenzie et al., 2017; Kang et al., 2018). Understanding the metabolic process of drugs is essential for successful drug discovery and development, and helps to optimize the stability of drugs, so as to optimize the half-life *in vivo*.

In order to reduce the risk caused by metabolic characteristics of candidate drugs, effective andreliable methods are needed to predict drug metabolism *in vitro*. Many experimental methods can be used to explore the metabolic process of drugs (Diao et al., 2016; Mackenzie et al., 2017). For example, fast LC-MS scans can be carried out to specifically detect predicted metabolites. However, experimental methods are still highly demanding in terms of equipment, expertise, cost, and time (Kirchmair et al., 2013). Therefore, it has great prospects to develop computational tools for predicting drug metabolism with lower cost and higher throughput than existing tools. Many different methodologies to predict metabolites or sites of metabolism have been reported recently. Various methods in predicting drug metabolism using in silico approaches have been reviewed (Fox and Kriegl, 2006; Gleeson et al., 2011; Zhang et al., 2011; Tan et al., 2017). However, most of these methods are limited to P450 catalytic reactions and represent only unstable sites, rather than predicting the actual metabolites formed.

Metabolic sites (SOMs) and metabolite structure are two main research directions of computer-aided metabolic prediction methods, which can provide decisive support and guidance for experimenters. SOM prediction methods usually have high prediction accuracy. The program MetaSite estimate the possibility of metabolic reactions at an atomic site using protein structure information, GRID-derived MIFs of protein, and ligand and molecular orbital calculations (Gabriele et al., 2005). The program SMARTCyp contains a pre-calculated energy reaction analysis table for density functional theory activation, where a large number of ligand fragments pass through CYP3A4 or CYP2D6 mediated transformation (Rydberg et al., 2010a; Rydberg et al., 2010b). A method called cypscore, in which 2400 CYP-mediated transformations and 850 literature compounds are used as data bases (Hennemann et al., 2010). However, most of these methods are limited to CYP450 catalytic reactions and can only predict unstable sites rather than metabolite structures. Furthermore, predicted SOMs are not identical to identifying the correct bioinformations that will occur at an atomic location, and they do not provide information about the type of reaction that will occur. Therefore, these limitations make it difficult to draw any quantitative conclusions about the metabolic possibilities of a molecule.

So far, only a few computational methods have been developed for predicting the structure ofmetabolites. Existing methods can be divided into two categories: expert rule-based anddescriptor-based. Rule-based approaches use data mining techniques. Large databases with data onmetabolism are used to extract generalized rules to determine the part of a molecule that undergoes metabolic alteration (Cariello et al., 2002). The ligand-based approach relies on the assumption that the metabolic fate of compounds is entirely determined by their chemical structure and properties. These methods include quantum mechanics methods. Descriptor-based methods to obtain an idea of the route of a drug through the metabolic system, the identification of the involved enzymes, and the reaction pathways is necessary (Livingstone, 2010). The program of Bioprint contains a database of most marketed drugs together with reference compounds and data from a wide variety of biological and *in-vitro* ADME assays, called the Biological fingerprint (Krejsa et al., 2003). Thus, the possible results of new compounds can be calculated by neighborhood relation and QSAR model. In the MetaDrug database (Ekins et al., 2006; Ekins et al., 2005b), metabolic reactions with substrates (including primary and secondary metabolites), xenobiotic reactions, and kinetic data on enzyme inhibition are stored. 317 molecules (parent drug and primary and secondary metabolite) were randomly selected from this database to build kernel-partial least squares models for metabolism rules (Embrechts and Ekins, 2007). Metabolite prediction is usually accomplished through a large set of transformation rules. Given the reactant, all rules are then matched to determine the site of metabolic instability. Expert systems such as METEOR (Testa et al., 2010; Button et al., 2015), META (Klopman et al., 1994; Talafous et al., 1994; Klopman et al., 1997), MetabolExpert (Darvas, 1988), RD-Metabolizer (Meng et al., 2017), MetaDrug (Korolev et al., 2003; Ekins et al., 2005a), and KnowItAll (Stouch et al., 2003) are based on these databases and provide a ranked list of most likely metabolites. In a study described by AstraZeneca (Scott et al., 2007), the substrates and reaction centers of the metabolite database were stored as fingerprints in two databases. Then the query molecule powders are compared with the two databases, and the proposed SOM is ranked by using the number of clicks as a weighted scheme. An approach called SyGMa based on the MDL metabolite database was developed (Ridder and Wagener, 2010). According to the corresponding rules of MDL metabolite data coding, the structure of possible metabolites is predicted, and probability scores are assigned to each metabolite, covering 70% of all known human metabolic reactions.

So far, one of the difficulties in predicting possible metabolites is that this task means identifying the reaction site (SOM) and the type of metabolic reaction correctly. Current methods for predicting metabolite structure tend to have high false-positive rates and can only be used for specific enzymes without covering all the metabolic enzymes involved in human reactions. In view of the above problems, we mainly designed a deep learning algorithm combined with drug metabolism characteristics.

**Figure 2:**
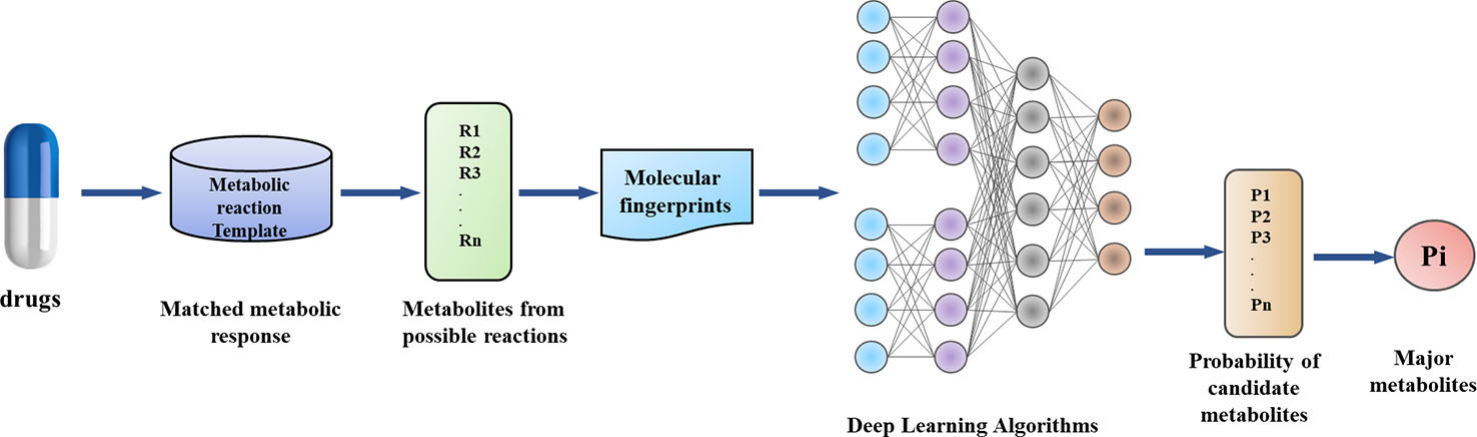
Metabolic reaction product prediction flow chart.

In this work, by combining metabolic reaction template and Deep Learning, we have established a model to predict the main metabolites of drugs (**Figure 2**). Our method has the following innovations: (1) Data enhancement strategy, which provides chemically reliable examples of negative reactions through the metabolic reaction template library; (2) the implementation and validation of a neural network-based model, which can obtain that some reaction modes are more or less likely to occur than other potential modes.

## Materials and Methods

### Data Collection and Processing

We collected metabolic reaction data from MDL Database (2011 edition). Here we used only human metabolic reactions with effective substrates and metabolites. The data were filtered to remove unreasonable structures, such as reactants and products containing R groups, free radicals, metal chelating, and structural errors, which could make it impossible to distinguish the reaction records of reaction sites. The pretreatment resulted in 7,380 reaction records, of which 74 reactions had only chiral changes. We randomly selected 300 response records from them as standby for external test sets.

### Generation of Metabolic Reactions Template Library

**Figure 3:**
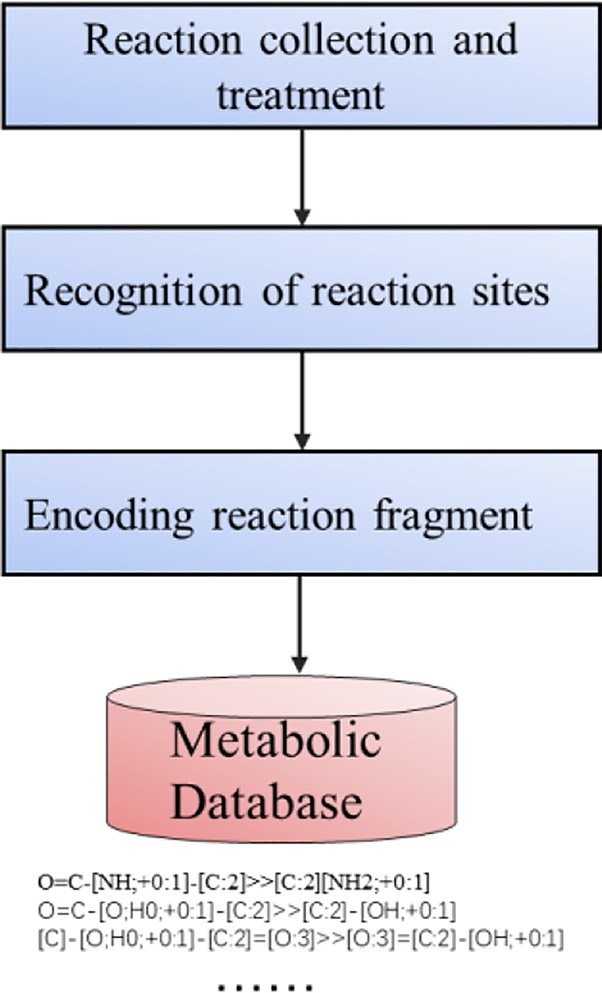
The generation of metabolic reactions template.

The process of constructing the metabolic reactions template library is shown in **Figure 3**. At present, the methods based on expert system mainly use the general metabolic rules deduced by experts to predict the structure of metabolites. However, this method has some drawbacks. The model needs to understand the influence of coding reaction functional groups. Such rules can not completely produce the desired response because the complete background of molecules is ignored. The remaining Non-coding functional groups of the molecule may affect or react competitively. So maybe even if the rules are matched, the ideal reaction product cannot be produced. Therefore, reaction rules need to be annotated with relevant information, such as functional groups, priority of reactions. However artificial code rules are time-consuming, laborious, and lack of internal ranking mechanism. Based on this, expert rules cannot be implemented on a large scale. Marwin H.S. (Law et al., 2009; Segler et al., 2018) has proved that in predicting the products of inverse synthesis reaction, the artificial extraction of reaction rules based on expert rules is far less effective than the algorithm which automatically judges the type of reaction according to the reactants and products in the deep learning model training. In order to construct a database of metabolic templates, we also adopted the heuristic driving algorithm of Law (Law et al., 2009).

**Table 1** shows the most common reaction types in the database. It can be seen that the most common metabolic reactions are amide hydrolysis, carboxylic acid hydrolysis, and hydroxylation of N, O, S atoms.

**Table 1:** The most common type of reactions and SMART fragments in the dataset.

Template SMART	Example
O = C-[NH;+0:1]-[C:2]> > [C:2][NH2;+0:1]	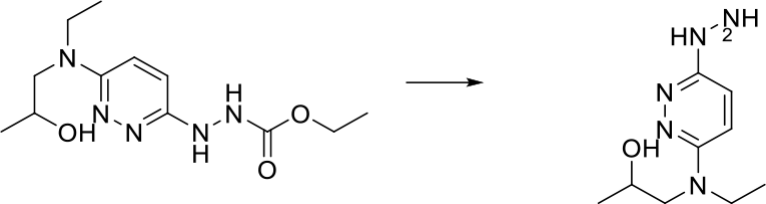
O = C-[NH;+0:1]-[c:2]> > [NH2;+0:1]-[c:2]	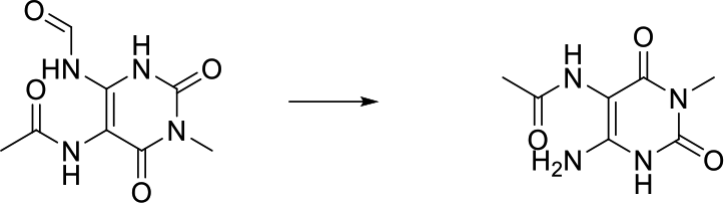
O = C-[O;H0;+0:1]-[C:2]> > [C:2]-[OH;+0:1]	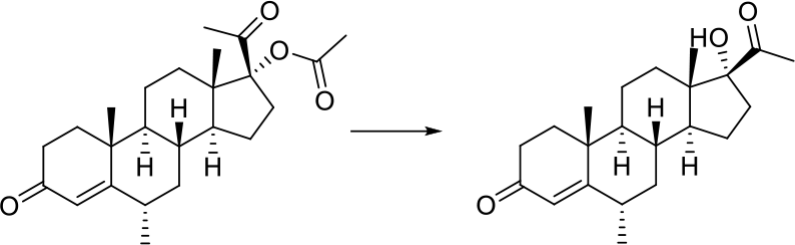
[C]-[O;H0;+0:1]-[C:2] = [O:3]> > [O:3] = [C:2]-[OH;+0:1]	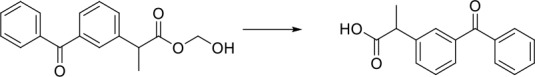
[C:1]-[N;H0;+0:2](-[C:3])-[C:4]> > [C:1]-[N+;H0:2](-[C:3])(-[C:4])	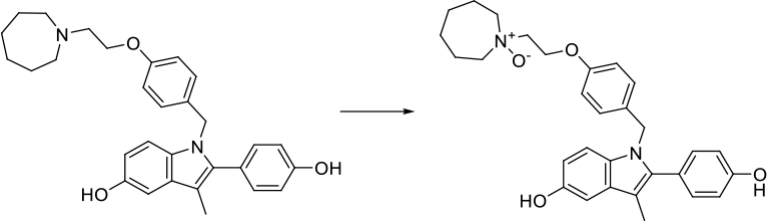
[c:1]-[S;H0;+0:2] [c:3]> > O = [S;H0;+0:2](-[c:1])-[c:3]	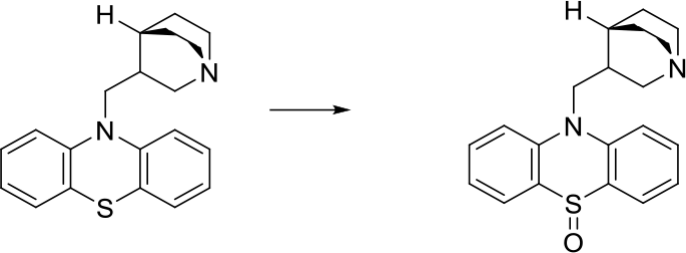
[C:1]-[CH2;+0:2]-[C:3]> > O-[CH;+0:2](-[C:1])-[C:3]	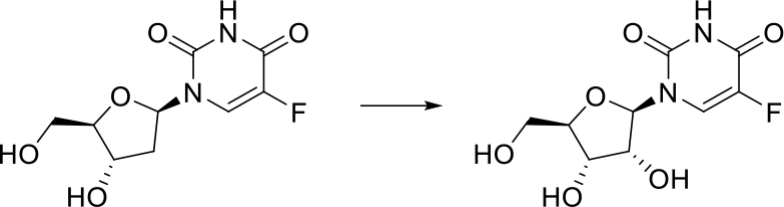
[c:1]:[n;H0;+0:2]:[c:3]> > [O-]-[n+;H0:2](:[c:1]):[c:3]	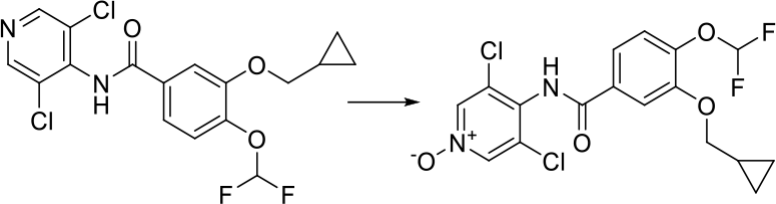
[c:1]:[cH;+0:2]:[c:3]> > O-[c;H0;+0:2](:[c:1]):[c:3]	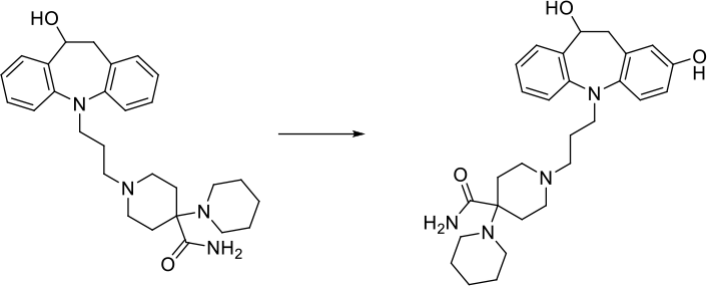
[C:1]-[S;H0;+0:2]-[C:3]> > O = [S;H0;+0:2](-[C:1])-[C:3]	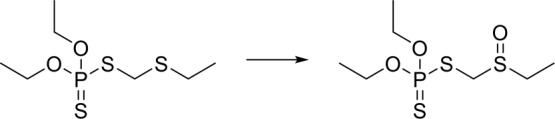

### Producing Candidate Metabolites

The above-mentioned metabolic reaction templates are stored in the database for subsequent production of positive and negative potential metabolites. For each atom mapping reaction in the dataset, the reaction center is defined by determining which product atoms are different from the corresponding reactant atoms. The reaction center is expanded to include the surrounding environment, and then other factors that play a role in the reaction is found out. Adjacent atoms are defined as non-hydrogen substituents, where high coverage is achieved at the expense of low specificity. Metabolic Templates are defined with SMART format strings encoding reaction centers. The reaction template generated in this way does not depend on manual extraction, marking, or sorting.

**Figure 4:**
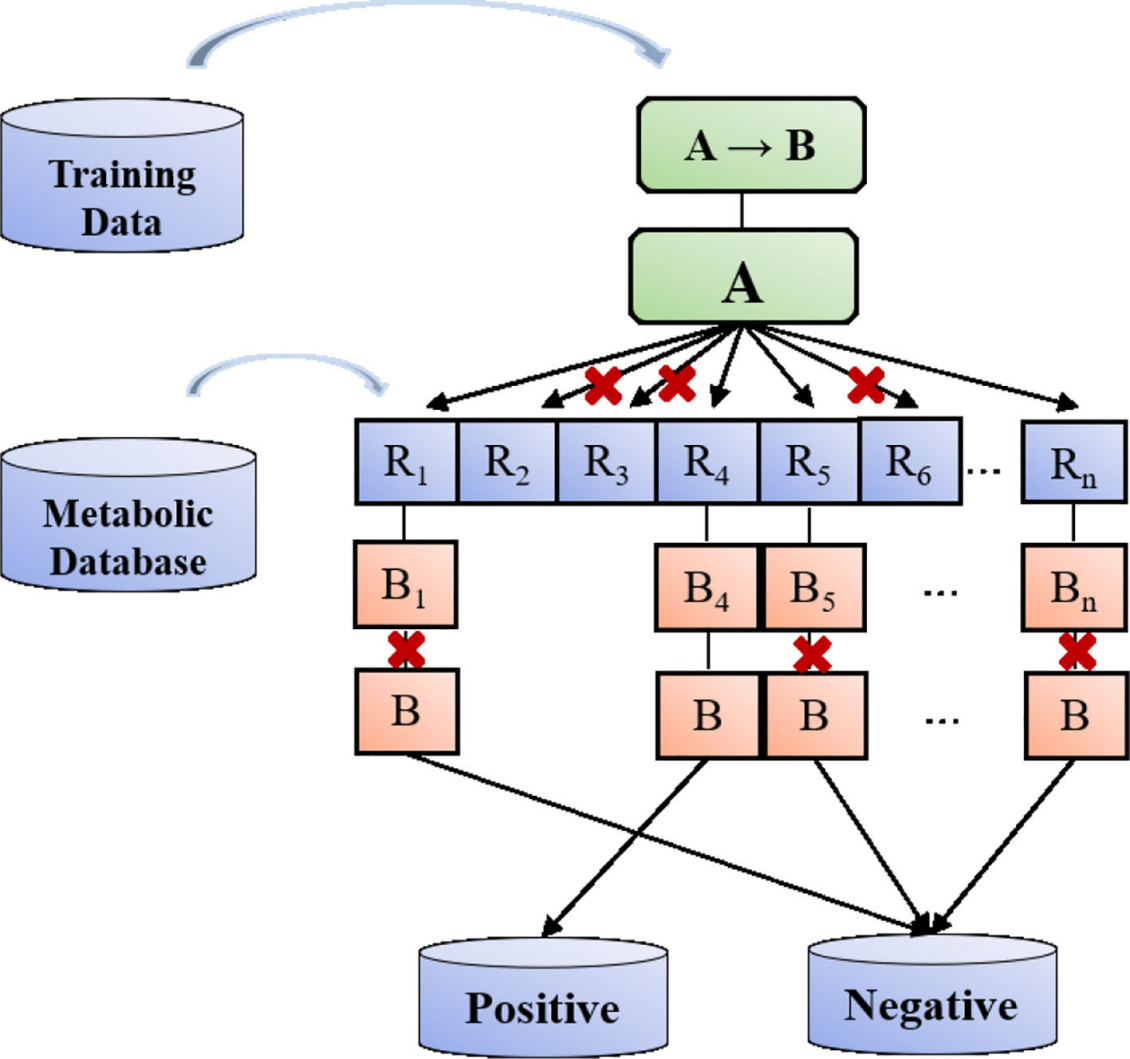
Flow chart for potential metabolites production.

As shown in **Figure 4**, we can match the reaction template of the metabolic database one by one and produce a large number of potential metabolic reaction products by using RDKit. Positive compounds are the products recorded in the database, and the rest are all negative products. This strategy can continuously produce negative products for later use.

### Model Training

For deep learning and supervised learning, we need to input eigenvalues. What we need to consider is using what molecular descriptors to characterize the whole metabolic process. Here we choose molecular fingerprints to describe the atomic and functional characteristics of metabolic reactions. The abstract representation of molecular fingerprints, which encodes molecular transformation into a series of vectors, makes it easier for molecules to compare with each other. If two molecules are similar, there must be many common fragments between them. Then molecules with similar fingerprints will have a high probability of being similar in 2D structures. Here we use RDKit to generate 1,024-dimensional Morgan molecular fingerprints. Molecular fingerprint ECFP is suitable for machine learning because it contains more molecular structure details. The metabolites generated above through the metabolic reaction template will be scored separately by Deep Learning model. Here we use Python library of Keras. The input layer consists of molecular fingerprints of products and reactants, with a total of 2,048 dimensions. One reaction corresponds to multiple potential metabolites. Thus, the input layer generates a matrix of 2,048 dimensions with *n* vectors. We use keras wrapper to realize each vector, that is, each individual potential metabolite is fully connected independently, which increases the ability of the model to achieve one-to-many and many-to-many. The probability of all potential reaction products is finally mapped out by the output layer activation function softmax, so that the total probability of all potential metabolites is 1. According to the score of the output layer, it is most likely to describe which metabolites actually exist. Deep neural network models are trained here to solve problems similar to classification problems: given hundreds of possible classes (potential metabolites), predicting real classes (recording reaction products), each metabolic reaction may correspond to multiple classifications. We use cross-entropy as the loss function during training. This objective function can be understood as the negative logarithm of probability allocated to the true class (true metabolites). During the training period, we use five-fold cross-validation to divide all the data sets into five parts, one of them is taken as the validation set without repetition, and the other four are used as training model of training set. Cross-validation can avoid over-fitting and under-fitting, and the final results are more convincing.

## Results and Discussion

### Accuracy of Prediction Results

Following the above steps, we cross-validated the model with five folds by using 200 epochs. The training set, validation set, test set segmentation was 7:1:2. The objective of the training period is to minimize the cross-entropy loss of classification, which is the natural logarithm of probability allocated to real metabolites. Considering that there may be more than one metabolic reaction product for a drug, we believe that the top ten predicted products may have more reference value. As shown in **Table 2**, the model achieves an average test set accuracy of 70% for Top-10 in the five-fold cross-validation. In addition, we also calculated the accuracy of Top-1, Top-3, and Top-6 rankings. Since our metabolites are generated automatically by the metabolic template obtained by the algorithm, as long as the template is matched, the reaction products can be formed. There is a problem with the explosion of potential metabolite combinations. It is a great challenge for the model to hit the product of the real reaction in the reaction product, but at the same time, the model can learn a lot of false product information because of the production of a large number of false metabolites, thus enhancing the learning ability.

**Table 2:** Prediction accuracies of the test set.

	Accuracy
Top-1	34%
Top-3	51%
Top-6	68%
Top-10	70%

**Figure 5:**
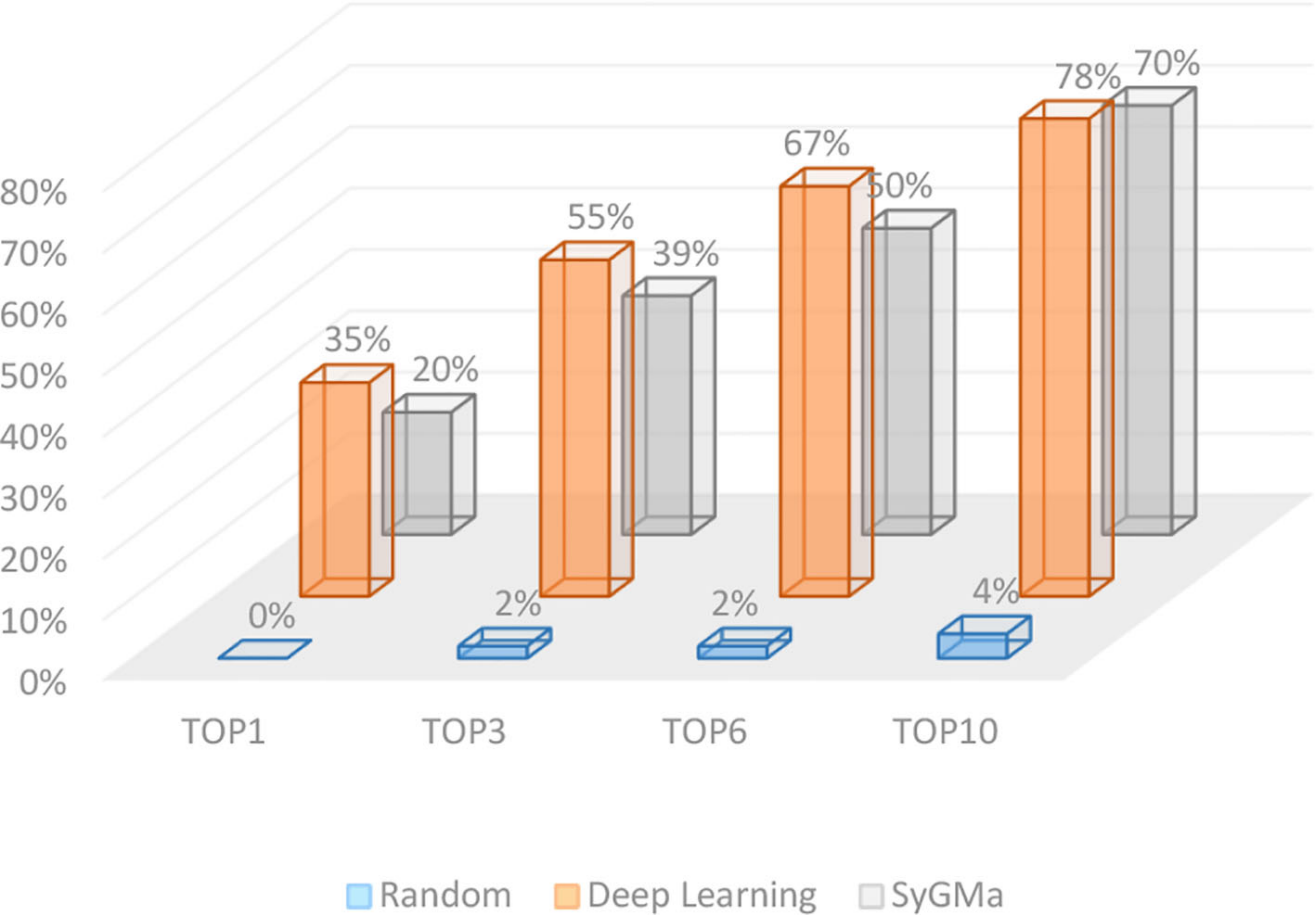
Comparison results on external test set.

Here, we conducted external tests on 300 reaction records that were not used for model training. It is also compared with the rule-based prediction method SyGMa. The accuracy rates of Top-1, Top-3, Top-6, and Top-10 are 35, 55, 67, and 78% respectively for our method (**Figure 5**). The accuracies of SyGMa for Top-1, Top-3, Top-6, and Top-10 are 20, 39, 50, and 70 respectively. The accuracy rates of our method are higher than SyGMa’s. The main reason is that SyGMa does not produce the correct metabolites in some reactions.

**Figure 6:**
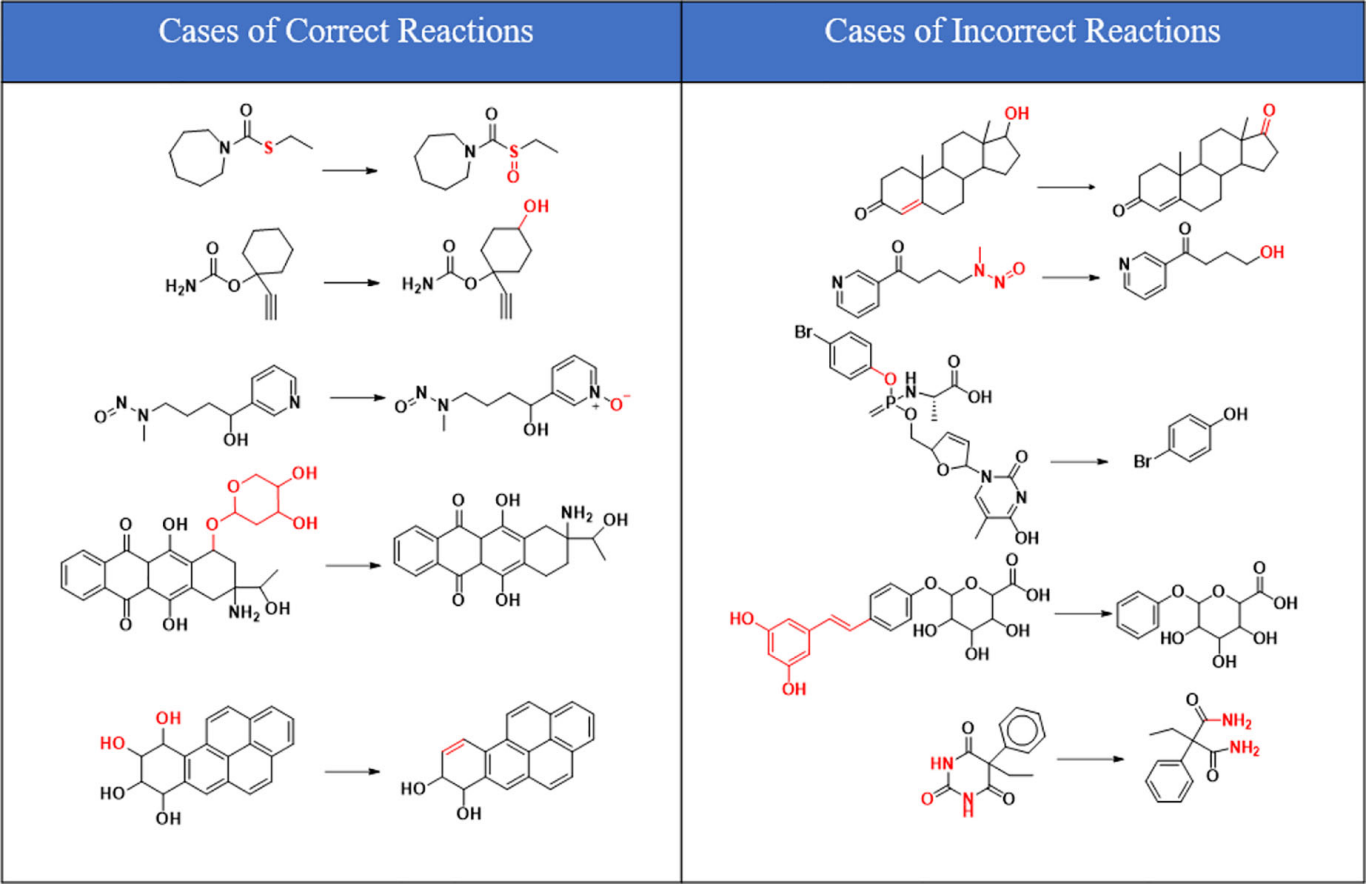
Reaction cases for correct and incorrect predictions.

As can be seen from **Figure 6**, correctly predicted metabolic reaction products by our method are common metabolic reactions, because these types of reaction samples account for the vast majority of the training set. Some of the metabolic reactions that cannot be correctly predicted are due to reaction types being uncommon with fewer occurrences in data sets, or because the reactants are too complicated and have multiple reaction sites. Furthermore, because usually only one metabolite of a compound is recorded in the reaction record, the Top-1 metabolite predicted by our method may not exactly be the recorded one, but it may still be one of the metabolites. Besides, in the reaction record involving multi-site and multi-step reactions, we can only predict a single-step metabolic reaction at one site. For our model, it is difficult for us to learn the changes in ring-opening and ring-closing reactions, because too much information is lost in those processes. It is difficult to characterize those metabolic processes only by the molecular fingerprints of reactants and products.

The amount of our data is small for Deep Learning to learn more information. The more reaction records that focused on a specific reaction, the more accurate the prediction of the reaction is. Thus we need to expand the data set for training. Next, we will collect more and more metabolic reactions from KEGG and other databases to train models, so as to improve the prediction accuracy of the models.

### Influences of Molecular Fingerprint Radius on the Results

**Figure 7:**
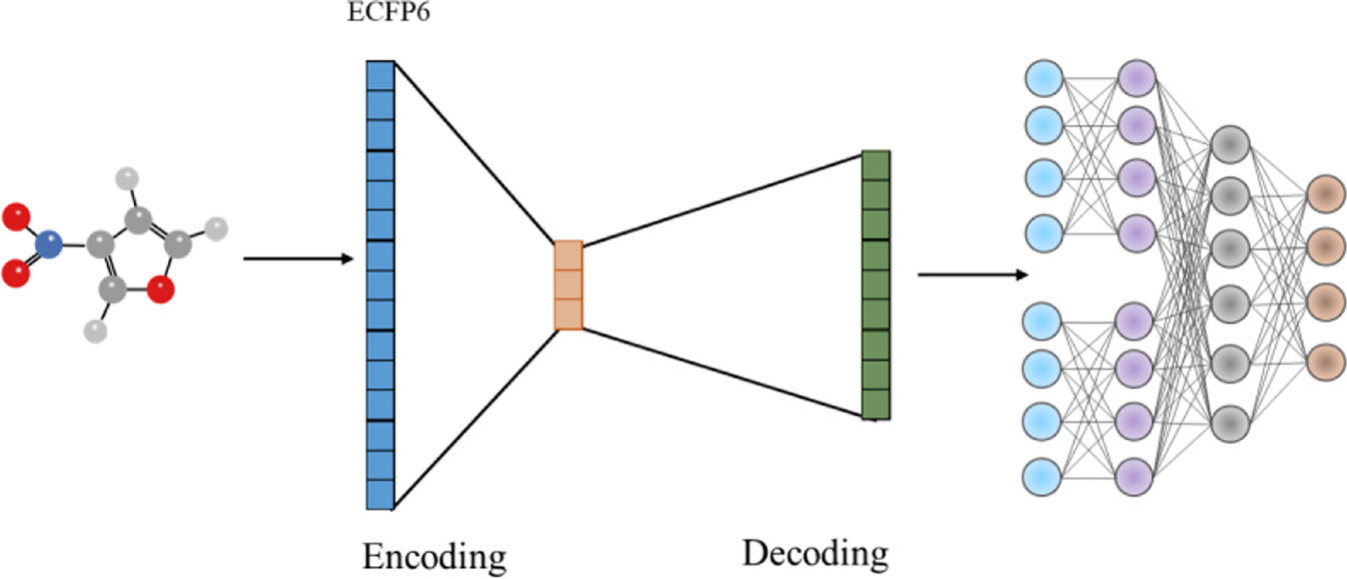
Flow chart of AutoEncoder combined with molecular fingerprint.

We retrieved Morgan molecular fingerprints with radius 3 from potential metabolic reaction products in training set and retrained them with AutoEncoder algorithm (**Figure 7**). Morgan molecular fingerprint with a radius of 3 is equivalent to ECFP6, which will contain more information about molecular fragments.

As shown in **Table 3**, increasing fingerprint radius did not improve the prediction accuracy of Top-1 and Top-3, but did improve the prediction accuracy of Top-10. The results suggested that increasing fingerprint radius can improve the accuracy of the model to a certain extent, and AutoEncoder algorithm can help improve the prediction ability of the model as well.

**Table 3:** Prediction accuracies at molecular fingerprint radius of 3.

	**Accuracy**
Top-1	32%
Top-3	51%
Top-6	68%
Top-10	81%

**Figure 8:**
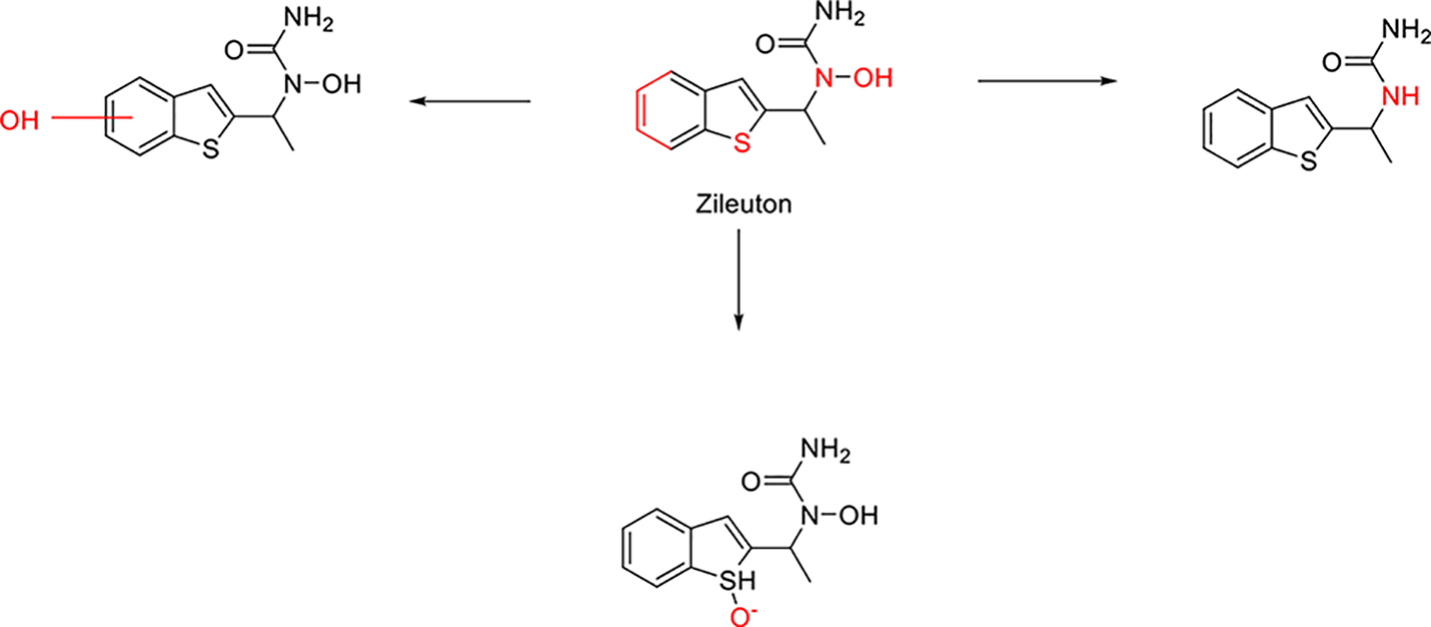
Metabolic pathways of Zileuton.

Here we take Zileuton as an example to analyze its prediction results of metabolites. It is an inhibitor of 5-lipoxygenase for the maintenance treatment of asthma. The main metabolic pathways of Zileuton are hydroxylation of benzene ring, oxidation of sulfur atoms on sulfur-containing heterocycles, and hydrolysis of nitrogen atoms on amide groups (Joshi et al., 2004) (**Figure 8** and **Table 4**).

**Table 4:** Top-10 predicted metabolites for Zileuton.

**Rank**	**Compounds**
1	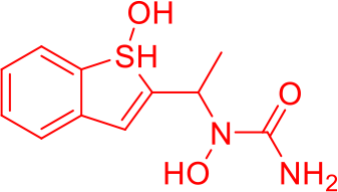
2	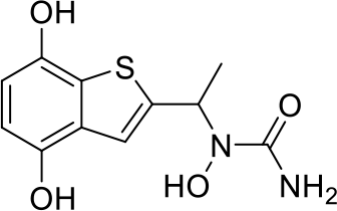
3	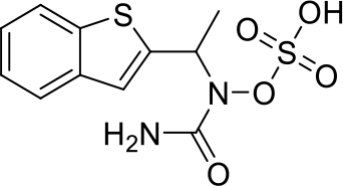
4	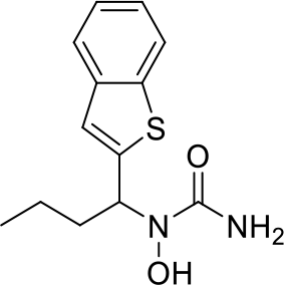
5	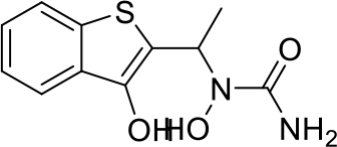
6	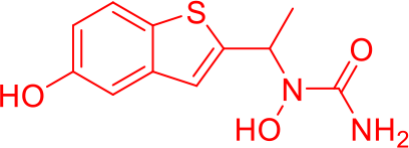
7	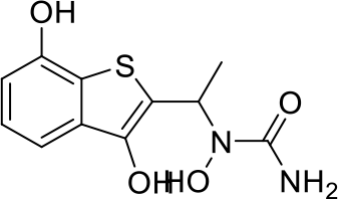
8	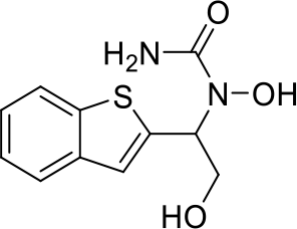
9	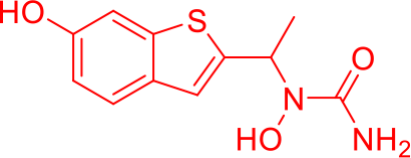
10	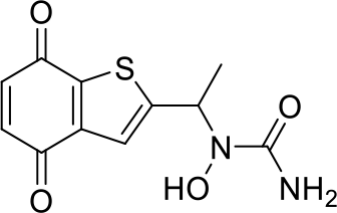

Here, three metabolites of zileuton were predicted correctly by our method, namely hydroxylation of the benzene ring and oxidation of sulfur atoms on sulfur-containing heterocycles. But our model has not predicted the hydrolysis of N atom of the side chain amide. The possible reason is that our training set has too little reactions to this type and the model has not adequately learned.

## Conclusion

In summary, we developed a deep learning based drug metabolites prediction algorithm to complement the experimental methods. By generating a broad coverage of metabolic reaction templates, we can generate a large number of potential metabolic reactants, and rank all metabolites by deep neural network algorithm to get the right metabolites ranked high. The accuracy of Top-1, Top-3, Top-6, and Top-10 in 300 external test sets with metabolic reactions is 35, 55, 67, and 78% respectively, which is significantly higher than that of random guess and the rule-based method SyGMa. Nevertheless, our method still has some limitations. It can rank the metabolites, but cannot give the probability of occurrence of metabolic sites. Besides, despite the relatively high prediction accuracy, it still has a high false-positive problem. To sum up, A approach of drug metabolites prediction based Deep learning was developed in this paper, which has certain predictive ability and can be used to provide some guidance information for researchers to improve the metabolic properties of lead compounds.

## Data Availability Statement

The raw data supporting the conclusions of this article will be made available by the authors, without undue reservation, to any qualified researcher.

## Author Contributions

DW conducted the research and wrote the paper. WL validated the model using known drugs and natural products. ZS helped collect the metabolic reaction data. LJ participated in building the metabolic reactions template library. JW tested the trained model. HL and SL designed and performed research, interpreted data, and approved the final manuscript.

## Funding

This work was supported by the National Key Research and Development Program (2016YFA0502304 to HL); the National Natural Science Foundation of China (grant 81825020 to HL, 81803437 to SL); the National Science & Technology Major Project “Key New Drug Creation and Manufacturing Program,” China (Number: 2018ZX09711002); the Fundamental Research Funds for the Central Universities, Special Program for Applied Research on Super Computation of the NSFC-Guangdong Joint Fund (the second phase) under Grant No.U1501501. SL is also sponsored by Shanghai Sailing Program (No. 18YF1405100). HL is also sponsored by National Program for Special Supports of Eminent Professionals and National Program for Support of Top-notch Young Professionals.

## Conflict of Interest

Author WL was employed by company Jiangzhong Pharmaceutical Co., Ltd.

The remaining authors declare that the research was conducted in the absence of any commercial or financial relationships that could be construed as a potential conflict of interest.

The reviewer MZ declared a past co-authorship with one of the authors HL to the handling editor.
